# Suppressed Fat Appetite after Roux-en-Y Gastric Bypass Surgery Associates with Reduced Brain μ-opioid Receptor Availability in Diet-Induced Obese Male Rats

**DOI:** 10.3389/fnins.2016.00620

**Published:** 2017-01-13

**Authors:** Mohammed K. Hankir, Marianne Patt, Jörg T. W. Patt, Georg A. Becker, Michael Rullmann, Mathias Kranz, Winnie Deuther-Conrad, Kristin Schischke, Florian Seyfried, Peter Brust, Swen Hesse, Osama Sabri, Ute Krügel, Wiebke K. Fenske

**Affiliations:** ^1^Department of Medicine, Integrated Research and Treatment Centre for Adiposity Diseases, University of LeipzigLeipzig, Germany; ^2^Department of Nuclear Medicine, University of LeipzigLeipzig, Germany; ^3^Helmholtz-Zentrum Dresden-Rossendorf, Research Site LeipzigLeipzig, Germany; ^4^Department of General and Visceral, Vascular and Paediatric Surgery, University of WürzburgWürzburg, Germany; ^5^Rudolf-Boehm Institute of Pharmacology and Toxicology, University of LeipzigLeipzig, Germany

**Keywords:** bariatric surgery, caloric-restriction, fat appetite, Brain μ-opioid receptors, positron emission tomography imaging

## Abstract

Brain μ-opioid receptors (MORs) stimulate high-fat (HF) feeding and have been implicated in the distinct long term outcomes on body weight of bariatric surgery and dieting. Whether alterations in fat appetite specifically following these disparate weight loss interventions relate to changes in brain MOR signaling is unknown. To address this issue, diet-induced obese male rats underwent either Roux-en-Y gastric bypass (RYGB) or sham surgeries. Postoperatively, animals were placed on a two-choice diet consisting of low-fat (LF) and HF food and sham-operated rats were further split into *ad libitum* fed (Sham-LF/HF) and body weight-matched (Sham-BWM) to RYGB groups. An additional set of sham-operated rats always only on a LF diet (Sham-LF) served as lean controls, making four experimental groups in total. Corresponding to a stage of weight loss maintenance for RYGB rats, two-bottle fat preference tests in conjunction with small-animal positron emission tomography (PET) imaging studies with the selective MOR radioligand [^11^C]carfentanil were performed. Brains were subsequently collected and MOR protein levels in the hypothalamus, striatum, prefrontal cortex and orbitofrontal cortex were analyzed by Western Blot. We found that only the RYGB group presented with intervention-specific changes: having markedly suppressed intake and preference for high concentration fat emulsions, a widespread reduction in [^11^C]carfentanil binding potential (reflecting MOR availability) in various brain regions, and a downregulation of striatal and prefrontal MOR protein levels compared to the remaining groups. These findings suggest that the suppressed fat appetite caused by RYGB surgery is due to reduced brain MOR signaling, which may contribute to sustained weight loss unlike the case for dieting.

## Introduction

The current obesity pandemic is primarily a consequence of the sudden widespread ease of access to palatable, energy-dense foods (Swinburn et al., 2011). Overeating in the face of plenty results from a complex interaction of emotional, cognitive and hedonic pressures which outweigh homeostatic processes in place to maintain stable body weight (Alsiö et al., 2012). A rich heritage of pharmacological studies has provided a picture of how opioidergic circuits interface with the brain systems that orchestrate feeding behavior (Bodnar, 2013). In rodents, agonists of the μ-opioid receptor (MOR) in particular have consistently been demonstrated to stimulate food intake when administered into various hypothalamic nuclei (Stanley et al., 1988), the central nucleus of the amygdala (Gosnell, 1988), ventromedial prefrontal and orbitofrontal cortices (Mena et al., 2011), and especially the striatum (Bakshi and Kelley, 1993). Here, acute administration of a selective MOR agonist into the nucleus accumbens potently stimulates, whereas a MOR antagonist inhibits fat intake in sated and fasted rats, respectively (Zhang et al., 1998). Moreover, intracerebral administration of antisense oligonucleotides against the MOR inhibits food intake and lowers body weight (Leventhal et al., 1996) while chronic administration of an irreversible MOR antagonist into the nucleus accumbens inhibits high-fat (HF) diet intake and prevents weight gain in rats (Lenard et al., 2010). These findings provide a rationale for the evaluation of MOR antagonists in weight loss studies performed on obese patients (Greenway et al., 2010; Ziauddeen et al., 2013).

Despite being by far the most efficacious treatment for obesity, little is known about how bariatric surgery affects the brain control of food intake to achieve sustained weight loss (Manning et al., 2015). Recently, a human positron emission tomography (PET) imaging study with the selective MOR radioligand [^11^C]carfentanil revealed widespread changes in brain MOR availability in obese subjects compared to lean, which were completely normalized after bariatric surgery (Karlsson et al., 2016). While a separate study reported modest changes in brain MOR availability in obese subjects after dieting (Burghardt et al., 2015), a direct comparison between these differing means of weight loss under standardized conditions has not been performed. This forms an important basis for gaining a better understanding of the central molecular underpinnings of successful long-term weight reduction unique to bariatric surgery. Additionally, it remains unclear whether alterations in brain MOR signaling following Roux-en-Y gastric bypass (RYGB), the most frequently employed bariatric surgical procedure, relates to the documented postoperative suppression in fat appetite in human (Kenler et al., 1990; Olbers et al., 2006; Thomas and Marcus, 2008) and animal (Zheng et al., 2009; le Roux et al., 2011; Shin et al., 2011b; Hao et al., 2016) studies. We therefore performed detailed fat intake and preference measurements in conjunction with *in vivo* small-animal [^11^C]carfentanil PET imaging studies in lean and diet-induced obese rats in comparison with animals that experienced identical weight loss from RYGB or chronic caloric-restriction. We then analyzed MOR protein expression in various brain regions by Western Blot.

## Materials and methods

### Animals

Sixteen male Wistar rats (RjHan:WI, outbred, Janvier, Le Genest-Saint-Isle, France) were used for our studies. Food and water were provided *ad libitum* unless otherwise stated. Animals were initially group housed and maintained on a 12-h light/dark cycle (lights on at 07:00 h) in facilities with an ambient temperature of 21–23°C and 40–60% humidity. All experiments were approved by the Institutional Animal Care and Use Committee at the Universität Leipzig with permission of the local government of Saxony (Regional Administrative Authority Leipzig, TVV 63/13, Germany). When indicated, diet induced obesity (DIO) was induced in 9 week old rats (*n* = 12) initially weighing approximately 350 g by feeding them for 5 weeks with a HF diet, which provides 58% of total energy as fat, 25.5% as carbohydrate, and 16.5% as protein (EF D12331, Ssniff GmbH, Soest, Germany). A separate group of rats always maintained on standard laboratory chow with 9% kcal derived from fat (RM1 diet; Ssniff GmbH, DE-59494, Germany) served as lean controls (*n* = 4).

### Abdominal surgeries and postoperative care

All abdominal surgical procedures were performed after an overnight fast by a bariatric surgeon according to a previously established protocol (Hankir et al., 2015; Seyfried et al., 2016). Briefly, animals were anesthetized with 5% isoflurane in 2 l/min oxygen and then maintained on 1.8–2.3% isoflurane in 0.5 l/min oxygen. Following induction of anesthesia, the abdominal wall was opened by a midline incision. For the RYGB procedure (*n* = 4), the jejunum was transected 15 cm aboral to the pylorus to create a 10 cm biliopancreatic limb. The proximal end was anastomosed to the ileum approximately 25 cm oral from the cecum, creating the common channel. The stomach was then transected 3 mm aboral to the gastroesophageal junction. At the proximal end, the gastric pouch (2–3% original stomach size) was anastomosed in an end-to-side fashion to the distal end of the small bowel forming the alimentary limb. At the distal end, the gastric remnant was closed using continues sutures. For the sham procedure (*n* = 12), the small bowel and the gastroesophageal junction were mobilized, and a 1 cm long gastrostomy was performed on the anterior wall of the stomach with subsequent closure. For postoperative analgesia, carprofen (5 mg/kg i.p.) was administered intraoperatively and then daily for 1 week. Postoperatively, all animals were singly-housed.

### Postoperative diet regimens

Standard laboratory chow with 9% kcal derived from fat (RM1 diet; Ssniff GmbH, DE-59494, Germany) mixed with water (wet diet) was given to all animals for 48 h following surgeries. Afterwards, the lean Sham-LF control group was maintained on standard laboratory chow, whilst the remaining DIO animals were postoperatively given a two-choice diet of standard laboratory chow and HF diet with 58% kcal derived from fat (EF D12331, Ssniff GmbH, DE-59494, Germany). DIO sham-operated animals were then randomly allocated into an *ad libitum* fed group (Sham-LF/HF group; *n* = 4) or a chronically food-restricted group body weight-matched to RYGB rats (Sham-BWM group; *n* = 4). To achieve this, animals from the Sham-BWM group were given approximately the same amount of HF diet as that consumed daily by the RYGB group in the early light phase, but the amount of LF diet was adjusted accordingly. Body weight and food intake were recorded daily for 12 weeks. This time-point after surgery corresponds to a stage of weight loss maintenance in our RYGB model (Hankir et al., 2015; Seyfried et al., 2016).

### Behavioral protocol for oral fat intake

During weeks 12–14 postoperatively, rats underwent 18-h two-bottle preference tests in which consumption of 5% concentration of an IntraLipid® fat emulsion (Fresenius Kabi, UK) was assessed. This concentration of fat emulsion was chosen based on previous observations (le Roux et al., 2011; and own unpublished data). After habituation with two water bottles for 72-h, *ad libitum* fed animals lacking side preferences had their food withdrawn and were then presented with two other bottles: one that contained 150 ml of water and the other 150 ml of 5% IntraLipid® solution on randomized sessions. Volumes ingested over 18 h were measured using an automated feeding-drinking monitoring system (TSE Systems GmbH, Bad Homburg, Germany). The preference ratio for fat emulsion was calculated as the ratio (%) between the volume ingested of fat emulsion and the total volume of fluid ingested.

### Synthesis of [^11^C]carfentanil

[^11^C]carfentanil was prepared according to a previously reported procedure (Dannals et al., 1985) applying the “loop method” (Wilson-Pérez et al., 2013). Specific activity as determined by high-performance liquid chromatography was 5.8 · 10^5^ MBq/nmol.

### Small-animal PET imaging protocol with the radioligand [^11^C]carfentanil

During weeks 15–17 postoperatively, animals underwent PET scanning under *ad libitum* fed conditions (just prior to feeding for the Sham-BWM group) using a dedicated small-animal PET/MR system (nanoScan®, Mediso Medical Imaging Systems, Budapest, Hungary) as previously described (Nagy et al., 2013). All PET scans were performed in the morning hours between 8 a.m. and 10 a.m. Rats were anaesthetized with 1.8% isoflurane in 0.5 l/min 60% oxygen/40% air mixture (Gas Blender 100 Series, MCQ instruments, Rome, Italy) before receiving a bolus intravenous injection of 41.6 ± 6.4 MBq [^11^C]carfentanil via the lateral tail vein. Simultaneous to tracer injection, a dynamic 35 min PET scan was initiated, during which animals were maintained at 37°C with a thermal bed system under isoflurane anesthesia. As animals' heads were too large to fit into the MR coil for subsequent MR imaging, for anatomical orientation a second static 15 min PET scan was performed immediately afterwards following intravenous bolus injection of approximately15 MBq [^18^F]- fluordeoxyglucose (FDG). The list-mode data for the [^11^C]carfentanil scan were rebinned into 28 frames (12 × 10, 6 × 30 s, 5 × 1 and 5 × 5 min) and iteratively reconstructed with 3D-ordered subset expectation maximization (OSEM), 4 iterations and 6 subsets using an energy window of 400–600 keV, coincidence mode of 1–5 and ring difference of 81. Cerebellum was selected as a reference region as a previous study performed on rats revealed minimal uptake of [^11^C]carfentanil in this brain region following intravenous administration (Quelch et al., 2014). [^11^C]carfentanil binding potential (BP_*ND*_) was calculated using the simplified reference tissue model (Lammertsma and Hume, 1996) for hypothalamus, thalamus, amygdala, striatum, cingulate cortex, insular cortex, prefrontal cortex and orbitofrontal cortex, again using the cerebellum as a reference region. This was performed after automatic co-registration of the [^18^F]-FDG PET images with a standard [^18^F]-FDG rat brain atlas using PMOD (v.3.6, PMOD Technology, Zurich, Switzerland) software. The final resolution of rendered PET images for analysis was 0.2 mm^3^.

### Western blot

At postoperative week 18, *ad libitum* fed rats were sacrificed and brains rapidly removed. Hypothalamus, striatum, prefrontal cortex and orbitofrontal cortex were carefully dissected and frozen immediately in liquid nitrogen. Protein was extracted from tissue (50 mg) in radioimmunoprecipitation assay (RIPA) buffer (40 μl/mg) after homogenization and incubation at 4°C for 1 h. Lysate was centrifuged for 15 min and supernatant collected. Sample (10 μg protein determined using a bicinchoninic acid (BCA) protein assay kit (ThermoFisher Scientific, Darmstadt, Germany)) was loaded into polyacrylamide gel, resolved by electrophoresis (125 V for 2 h) and transferred onto a nitrocellulose membrane using a semi-dry method (75 mA for 1 h). Membranes were blocked with 3% bovine serum albumin (BSA) for 30 min and after washing were incubated overnight at 4°C with rabbit anti-rat MOR monoclonal primary antibody (AbCam, Cambridge, UK) diluted 1/1000 in 3 % BSA. Membranes were then incubated for 1 h at room temperate with goat anti-rabbit IgG secondary antibody conjugated to horseradish peroxidase (Cell Signaling Technology, Leiden, Netherlands) diluted 1/3000 in 3% BSA. Bands were visualized using an enhanced chemiluminescence (ECL) kit (Biozym Scientific GmbH, Oldendorf, Germany) and quantified using the rolling method with GeneSnap software (v7.12, SynGene, Cambridge, UK). As a loading control, membranes were stripped with 0.2 N NaOH and β-actin was measured using rabbit anti-rat β-actin monoclonal primary antibody (Sigma Aldrich Chemie GmbH, Taufkirchen, Germany) diluted 1/500 in 3% BSA and then the same secondary antibody as for the MOR.

### Statistics

Data sets were analyzed for statistical significance using GraphPad Prism software (v.7.02, GraphPad Software Inc., La Jolla, CA). One-way and two-way ANOVAs corrected for multiple comparisons with Tukey's *post-hoc* tests were used as required.

## Results

### RYGB surgery reduces body weight, HF diet intake, and preference in diet-induced obese male rats

**Figure 1:**
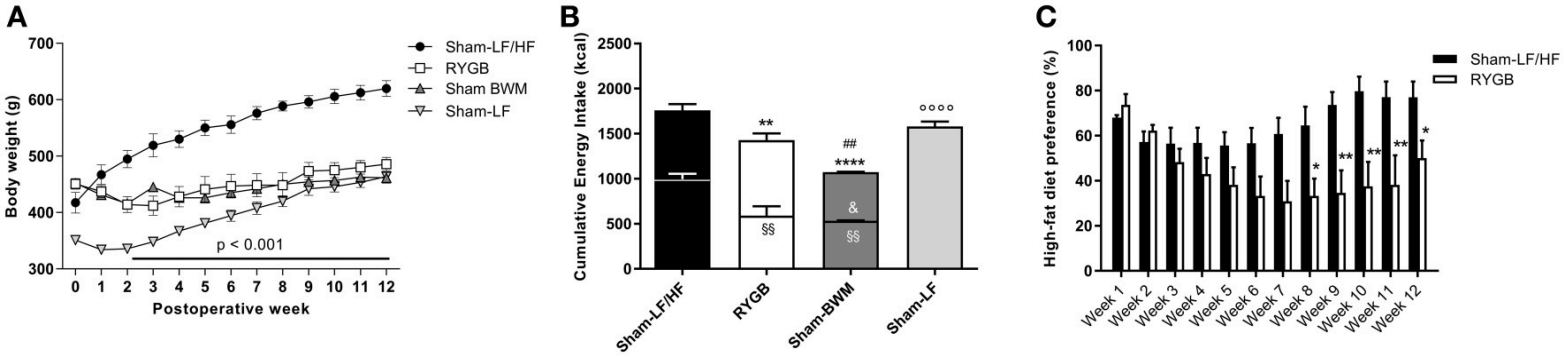
**RYGB surgery reduces body weight, food intake and HF diet preference in diet-induced obese male rats**. High-fat (HF) diet-induced obese rats were split into 3 groups: Roux-en-Y gastric bypass-operated and then maintained on a choice diet (RYGB), sham-operated and then maintained on a choice diet (Sham-LF/HF) and sham-operated and then body weight-matched to RYGB rats (Sham-BWM). A separate group of never obese sham-operated rats always maintained on a LF diet (Sham-LF) was also added as lean controls. **(A)** Shows weekly body weights and **(B)** total cumulative energy intake over 12 weeks. In **(A)**, horizontal bar denotes significance between the Sham-LF/HF group and the remaining groups. In **(B)**, lower bar segments represent energy in kcal consumed from HF food and upper bar segments represent energy in kcal consumed from LF food. **(C)** Weekly HF diet preference was calculated by dividing the amount of HF diet consumed with the total amount of diet (HF and LF) consumed and expressed as percentage (%). Data are presented as mean ± SEM. *n* = 4 per treatment group. ^*^p < 0.05, ^**^p < 0.01 and ^****^p < 0.0001 vs. Sham-LF/HF, ^*##*^*p* < 0.05 vs. RYGB and ^◦◦◦◦^*p* < 0.0001 vs. Sham-LF for total energy intake; ^§§^*p* < 0.01 vs. Sham-LF/HF for HF food intake; ^&^*p* < 0.05 vs. RYGB for LF food intake.

To confirm that our RYGB rat model recapitulates the human phenotype observed after RYGB, body weight, food intake and food preference were regularly measured postoperatively compared to obese and lean control groups. With respect to body weight (Figure 1A); preoperative values were similar between Sham-LF/HF (418 ± 18.4 g), RYGB (451 ± 9.4 g), and Sham-BWM (452 ± 4.4 g) groups but was significantly less (*p* < 0.01) for the Sham-LF group (351 ± 3.4 g). From postoperative week 2, body weights started to significantly diverge (*p* < 0.0001) between Sham-LF/HF and post-DIO weight loss groups (RYGB and Sham-BWM) which continued for the duration of the 12 week recording period. Body weights of Sham-LF rats steadily increased during this period so that they were similar to post-DIO weight loss groups by week 12, but significantly less (*p* < 0.0001) than Sham-LF/HF rats.

Concerning total food consumption (Figure 1B), cumulative energy intake over the 12 week recording period was considerably lower (*p* < 0.01) for RYGB rats (1428 ± 71.5 kcal) compared to Sham-LF/HF rats (1760 ± 39.7 kcal), but higher (*p* < 0.01) compared to Sham-BWM rats (1074 ± 8.8 kcal). The similar body weights despite different food intakes between RYGB and Sham-BWM rats has previously been attributed to differences in energy expenditure (Hankir et al., 2015). When focusing on HF diet intake alone in groups given access (restricted or free) to it (Figure 1B), consumption was markedly less (*p* < 0.01) for RYGB (590 ± 105.5 kcal) and Sham-BWM rats (535 ± 3.5 kcal) compared to Sham-LF/HF rats (996 ± 59.3 kcal). The amount of HF diet given to Sham-BWM rats was ensured to be similar (*p* = 0.85) to that consumed by RYGB rats. Consequently, to cause and sustain weight loss, Sham-BWM rats were given significantly less (*p* < 0.05) LF diet (539 ± 5.3 kcal) than that consumed by RYGB rats (839 ± 75.2 kcal).

With respect to dietary preference in groups with *ad libitum* access to a choice diet (Figure 1C), RYGB rats showed an overall reduced preference for HF diet relative to Sham-LF/HF rats over the 12 week recording period [main effect of time *F*_(11, 72)_ = 2.24; *p* = 0.028, main effect of treatment *F*_(1, 72)_ = 50.9; *p* < 0.0001, and interaction *F*_(11, 72)_ = 2.40; *p* = 0.013]. This difference started at postoperative week 8 (64.6 ± 8.4% for Sham-LF/HF rats vs. 33.3 ± 7.7% for RYGB rats; *p* < 0.05) and was maintained until postoperative week 12 (77.1 ± 7.1% for Sham-LF/HF rats vs. 50.0 ± 7.9% for RYGB rats; *p* < 0.05).

### RYGB surgery suppresses appetite for high concentration fat emulsion

**Figure 2:**
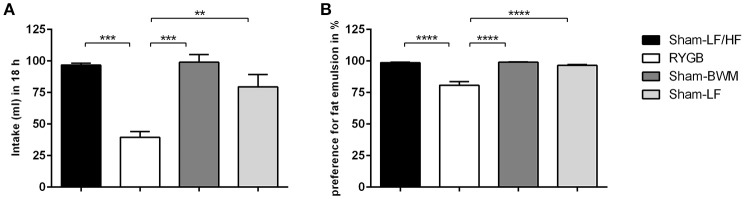
**RYGB surgery suppresses intake and preference for high concentration lipid emulsions in diet-induced obese male rats**. Two-bottle fat preference tests were performed on Sham-LF/HF, RYGB, Sham-BWM and Sham-LF rats during postoperative weeks 12–14. Animals were presented with two pre-measured bottles: one that contained water and the other HF emulsion containing 5% Intralipid®. **(A)** Volumes ingested were measured over the course of 18 h and **(B)** fat preference was calculated by dividing the volume of 5% IntraLipid® consumed by that of the total volume of liquid (water and 5% Intralipid®) consumed in the session and expressed as percentage (%). Data are presented as mean ± SEM. *n* = 4 per treatment group. ^**^p < 0.01, ^***^p < 0.001, ^****^p < 0.0001.

In order to study the effects of weight loss from RYGB and chronic caloric-restriction on fat appetite in more detail, we then performed two-bottle fat preference tests with a high concentration (5%) IntraLipid® emulsion on RYGB and Sham-BWM groups compared to obese (Sham-LF/HF) and lean (Sham-LF) control groups. The RYGB group presented with a strikingly lower (*p* < 0.01) intake of 5% IntraLipid® relative to the remaining groups (Figure 2A). Intake of 5% Intralipid® over 18 h was 40 ± 4.6 ml for RYGB, 96 ± 1.5 ml for Sham-LF/HF, 99 ± 6.2 ml for Sham-BWM and 80 ± 9.7 ml for Sham-LF rats. When calculated as preference over water (Figure 2B), the difference between the RYGB group and the remaining groups became more apparent (*p* < 0.0001). Preferences for high concentration fat emulsion were 80 ± 2.8% for RYGB, 98 ± 0.5% for Sham-LF/HF, 99 ± 0.3% for Sham-BWM, and 97 ± 0.7% for Sham-LF rats.

### RYGB surgery reduces brain MOR availability

**Figure 3:**
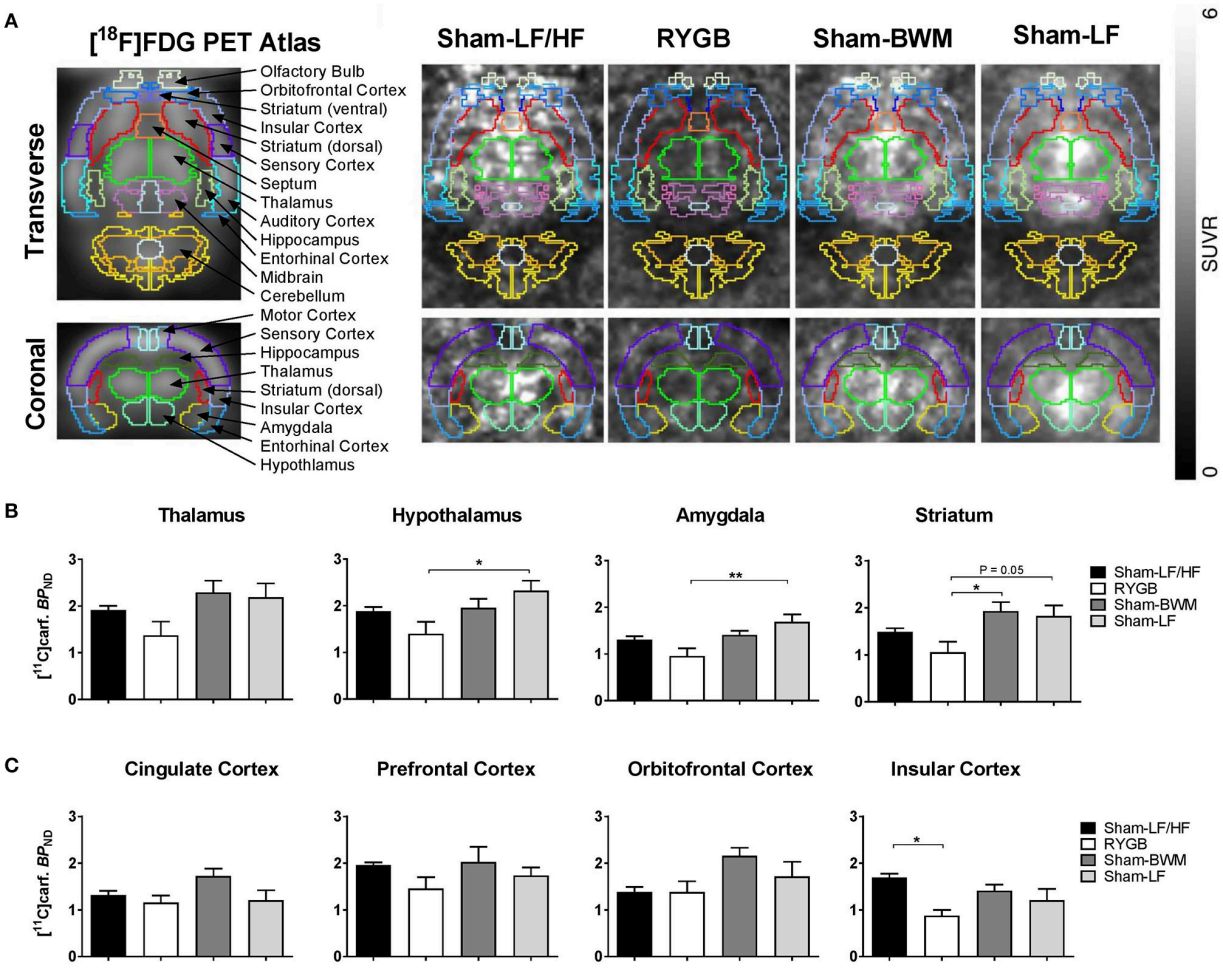
**Reduced brain MOR availability at a stage of weight loss maintenance after RYGB in diet-induced obese male rats**. During postoperative weeks 15–17, *ad libitum* fed Sham-LF/HF, RYGB, Sham-BWM and Sham-LF rats underwent small-animal PET imaging. Animals were anesthetized with isoflurane and received the selective μ-opioid receptor (MOR) radioligand [^11^C]carfentanil intravenously at the onset of PET scanning for 35 min. **(A)** (Left) standard [^18^F]-FDG rat brain PET atlas used to align PET images with brain masks superimposed and labeled. (Right) representative PET images of Sham-LF/HF, RYGB, Sham-BWM and Sham-LF rat brains depicted as a standardized uptake value (SUV) ratio with cerebellum used as reference region based on frames 20–35 min. [^11^C]Carfentanil binding potential ([^11^C]Carf. BP_*ND*_) was calculated for individual **(B)** subcortical and **(C)** cortical regions of interest. Data are presented as mean ± SEM. *n* = 4 per treatment group. ^*^p < 0.05, ^**^p < 0.01.

Having found differential effects of weight loss from RYGB surgery and chronic caloric-restriction on fat appetite, we then sought to determine the potential role of brain MORs for this difference in feeding behavior. We therefore performed small-animal PET imaging with the MOR radioligand [^11^C]carfentanil which provides a measure of brain MOR availability *in vivo* (Burghardt et al., 2015; Karlsson et al., 2016). On the morning of scans, Sham-LF/HF rats were significantly heavier (*p* < 0.0001) compared to the remaining groups. Sham-LF/HF rats weighed 653 ± 3.3 g, RYGB rats weighed 508 ± 17.3 g, Sham-BWM rats weighed 496 ± 14.1 g and Sham-LF rats weighed 501 ± 18.5 g. After scanning, inspection of PET images revealed a widespread reduction in [^11^C]carfentanil uptake in the brains of RYGB rats compared to the remaining groups (Figure 3A). PET data analysis for all subcortical (thalamus, hypothalamus, striatum, and amygdala) and cortical (prefrontal cortex, orbitofrontal cortex, cingulate cortex, and insular cortex) regions analyzed are shown in Figures 3B,C, respectively. In subcortical regions, [^11^C]carfentanil binding potential (BP_*ND*_) was significantly lower in RYGB rats relative to Sham-LF rats in the hypothalamus (*p* < 0.05), and amygdala (*p* < 0.01) and tended to be lower in the striatum (*p* = 0.06). [^11^C]carfentanil BP_*ND*_ was also significantly lower in RYGB animals relative to Sham-BWM animals in the striatum (*p* < 0.05). In cortical regions, [^11^C]carfentanil BP_*ND*_ was only significantly lower in RYGB rats relative to Sham-LF/HF rats in the insular cortex (*p* < 0.05).

### RYGB surgery reduces striatal and prefrontal MOR protein expression

**Figure 4:**
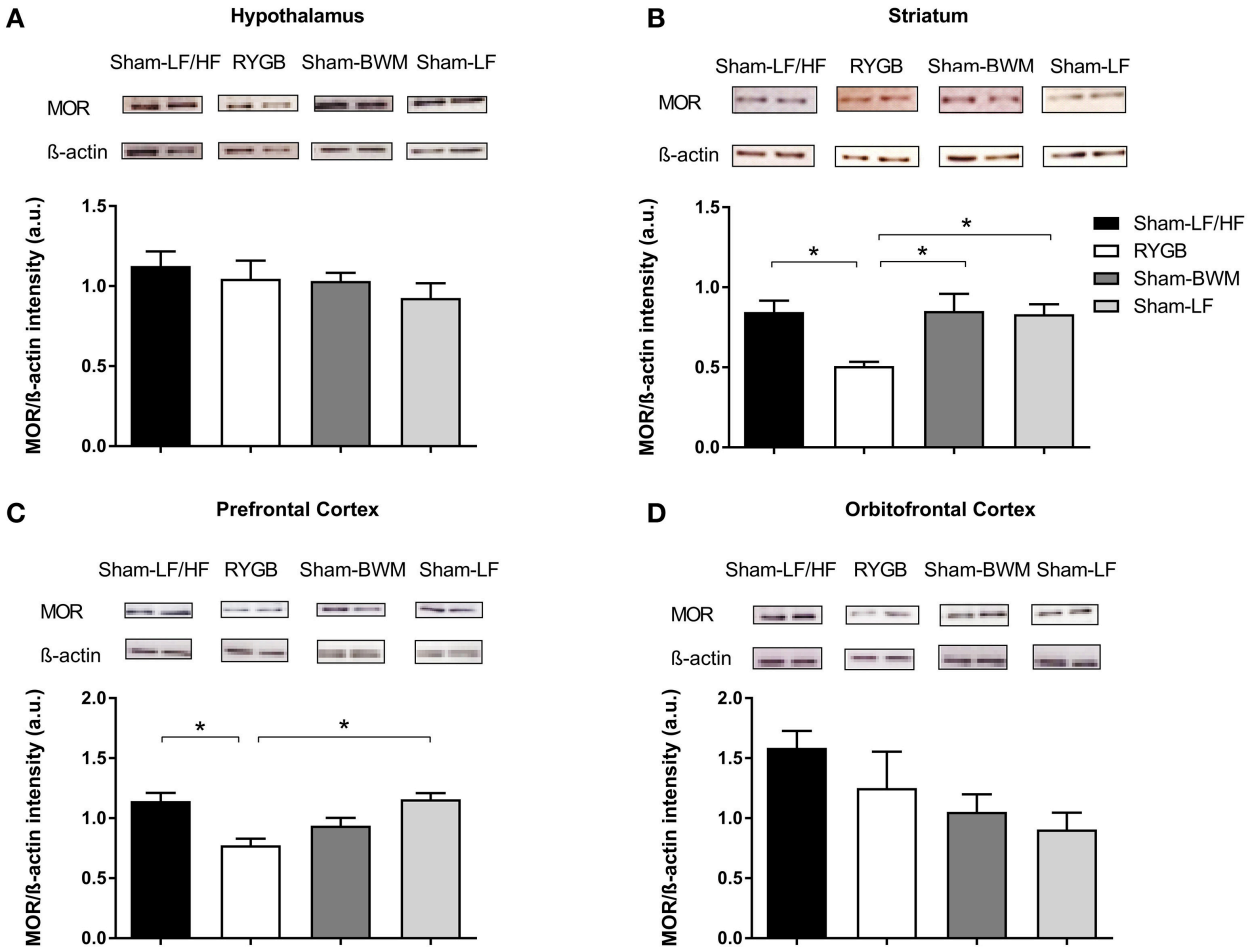
**Downregulation of striatal and prefrontal MOR protein levels at a stage of weight loss maintenance after RYGB in diet-induced obese male rats**. At postoperative week 18, *ad libitum* fed Sham-LF/HF, RYGB, Sham-BWM, and Sham-LF rats were sacrificed and brains were collected for Western Blot analysis. Representative blots for MOR and β-actin in **(A)** hypothalamus, **(B)** striatum, **(C)** prefrontal cortex, and **(D)** orbitofrontal cortex for each group are presented. In **(A–D)**, MOR protein expression was quantified by normalizing band signal intensity to that of β-actin. Data are presented as mean ± SEM. *n* = 3/4 per treatment group. ^*^p < 0.05.

Considering the PET data showing widespread reductions in brain MOR availability after RYGB, we next measured MOR protein expression in brain regions involved in the opioidergic control of feeding including the hypothalamus (Stanley et al., 1988), striatum (Bakshi and Kelley, 1993; Zhang et al., 1998; Lenard et al., 2010), orbitofrontal cortex (Mena et al., 2011), and prefrontal cortex (Mena et al., 2011) in our experimental groups by Western Blot. Immunoblots revealed a distinct 47 kDa band corresponding to the molecular weight of the MOR (Figure 4). Analysis of band intensity normalized to β-actin revealed lower values for the RYGB group relative to the remaining groups (*p* < 0.05) in the striatum (Figure 4B), and for the RYGB group relative to Sham-LF/HF (*p* < 0.05) and Sham-LF (*p* < 0.05) groups in the prefrontal cortex (Figure 4C).

## Discussion

Brain MORs are in prime position to be a molecular target of RYGB due to their prominent role in regulating hedonic feeding (Bodnar, 2013). In accordance with previous human (Kenler et al., 1990; Olbers et al., 2006; Thomas and Marcus, 2008) and rodent (Zheng et al., 2009; le Roux et al., 2011; Shin et al., 2011b; Hao et al., 2016) studies, we demonstrated that RYGB reduces HF intake and preference using an established rat model (Hankir et al., 2015; Seyfried et al., 2016). We found that this was associated with widespread reductions in brain MOR availability, particularly in the striatum. Furthermore, levels of MOR protein in the striatum as well as in the prefrontal cortex were downregulated after RYGB, which is consistent with the known stimulatory effects of MOR agonism in these areas on fat intake (Bakshi and Kelley, 1993; Mena et al., 2011). In contrast, no changes in feeding behavior or brain MOR signaling were observed in chronically food restricted Sham-BWM animals. Reduced brain MOR signaling, therefore, may underlie the postoperative suppression in HF food intake, contributing to sustained weight loss caused by RYGB, unlike the case for chronic food restriction.

Previous studies performed on rats support a key role for nucleus accumbens MORs in the “liking” of palatable foods (Richard et al., 2013). In this context, and in line with the findings from the present study of reduced striatal MOR levels after RYGB, patients and rats demonstrate measures of diminished HF food liking postoperatively (Shin et al., 2011b; Ochner et al., 2012). However, patients and rats also show the same response following chronic caloric-restriction (Epstein et al., 1989; Shin et al., 2011a). Considering that we did not find alterations in striatal MORs in Sham-BWM rats, it appears that changes in taste reactivity following weight loss caused by chronic caloric-restriction may be dissociated from striatal MOR signaling.

There is considerable overlap between the effects of RYGB and reduced brain MOR signaling on brain function and behavior. For instance, in human fMRI studies both RYGB and MOR antagonist treatments decrease striatal blood oxygenation level dependent signal when subjects are presented images of HF foods (Cambridge et al., 2013; Scholtz et al., 2014) and in rat macronutrient studies, both RYGB and MOR antagonist treatments suppress fat intake when carbohydrate, fat and protein diets are presented simultaneously (Marks-Kaufman and Kanarek, 1990; Wilson-Pérez et al., 2013). In addition, patients who take opioids before RYGB tend to use higher amounts after surgery (Raebel et al., 2013) and a recent investigation revealed that male RYGB rats have markedly higher morphine self-administration rates on a fixed ratio schedule of reinforcement compared to obese, chronically caloric-restricted and lean counterparts (Biegler et al., 2016). These latter findings strongly support those of the present study of reduced brain MOR signaling after RYGB, which may predispose to a compensatory increase in opioid intake as is typically observed with brain receptor downregulation/desensitization during drug tolerance.

The reduced brain MOR availability following RYGB is body weight independent and likely a consequence of altered gut-brain communication. Circulating levels of the orexigenic stomach hormone ghrelin have been previously reported to be decreased in patients (Cummings et al., 2002) and rodents (Shin et al., 2010) after RYGB. Considering that systemic administration of ghrelin increases brain MOR mRNA expression in rats (Skibicka et al., 2012), it is possible that the RYGB-mediated lowering in plasma ghrelin contributes to reduced brain MOR levels postoperatively. Profound changes in gut microbiota also take place after RYGB in humans and rodents (Furet et al., 2010; Liou et al., 2013; Shao et al., 2016). Interestingly, secreted proteins from gut *E. coli* have been found to influence brain opioidergic feeding circuits (Breton et al., 2016) and may too underlie altered brain MOR signaling after RYGB. Future mechanistic studies can address these issues in more detail.

It was recently reported in human [^11^C]carfentanil PET imaging studies that dieting exerts modest effects (Burghardt et al., 2015) whereas bariatric surgery causes a widespread increase in brain MOR availability in obese subjects (Karlsson et al., 2016). With respect to the study of Burghardt et al., [^11^C]carfentanil BP_*ND*_ in various brain regions was not significantly different between fasted obese individuals before (BMI 38) and after (BMI 32) weight loss caused by 4 months of dieting. These findings are comparable with those made in the present study with the Sham-LF/HF and Sham-BWM animals. With respect to the study of Karlsson et al. [^11^C]carfentanil BP_*ND*_ in thalamus, ventral striatum, dorsal striatum, amygdala, insular cortex, and orbitofrontal cortex in mildly fasted obese individuals was markedly *increased* 6 months after bariatric surgery (BMI 31) relative to the preoperative state (BMI 40). In contrast, here we report a *reduction* in MOR availability in these brain regions postoperatively. This finding was robust, attaining statistical significance despite the low sample size. A possible explanation for the discrepant findings between the present study and the study of Karlsson et al. is that the timing of our scans corresponded to a late postoperative stage of weight loss maintenance after RYGB, whereas the subjects in the study of Karlsson et al. were scanned at an early stage of active weight loss. We also exclusively report findings from male rats after RYGB surgery, whereas Karlsson et al. performed PET analysis on a mixed population of female patients whom underwent two different bariatric procedures (sleeve gastrectomy and RYGB). Indeed the female sex hormones estrogen and progesterone both have been shown to influence brain MOR expression (Hammer et al., 1994).

A limitation of radioligand PET studies in general is that binding potentials do not provide a strict measure of receptor density. In the case for [^11^C]carfentanil, binding to the MOR can be reduced as a result of competitive displacement by endogenous opioids (Burghardt et al., 2015). However, we found that in the striatum and prefrontal cortex at least, RYGB downregulates MOR protein expression which may underlie the reduced [^11^C]carfentanil binding in these regions. Future cerebral microdialysis studies can reveal whether RYGB influences brain opioid release in rats (Difeliceantonio et al., 2012).

In summary, we report that suppressed fat appetite at a stage of weight loss maintenance after RYGB in male rats is associated with reduced brain MOR availability. Bariatric surgery may uniquely target various anatomically and molecularly discrete brain feeding circuits (Haahr et al., 2015) at early and late time points postoperatively (Mumphrey et al., 2016). Thus, treatments which can mimic the dynamic neurochemical signature of RYGB might effectively improve feeding behavior in the long-term, causing and sustaining significant weight loss in obese individuals.

## Author contributions

Conceptualization, MKH and WF; investigation, MP, MK, WD, MHK, FS, UK, KS; formal analysis and visualization, GB, PB, SH, OS; writing original draft, MKH and WF; writing review and editing, MHK and WF; funding acquisition and project administration, WF; resources, UK and WF; project design and supervision, WF.

### Conflict of interest statement

The authors declare that the research was conducted in the absence of any commercial or financial relationships that could be construed as a potential conflict of interest.
